# Higher expression of human kallikrein 10 in breast cancer tissue predicts tamoxifen resistance

**DOI:** 10.1038/sj.bjc.6600323

**Published:** 2002-06-05

**Authors:** L-Y Luo, E P Diamandis, M P Look, A P Soosaipillai, J A Foekens

**Affiliations:** Department of Pathology and Laboratory Medicine, Mount Sinai Hospital, Toronto, ON, M5G 1X5, Canada; Department of Laboratory Medicine and Pathobiology, University of Toronto, Toronto, ON, M5G 1L5, Canada; Division of Endocrine Oncology, Department of Medical Oncology, Rotterdam Cancer Institute (Daniel den Hoed Kliniek) and University Hospital Rotterdam, Rotterdam, The Netherlands

**Keywords:** human kallikrein 6, human kallikrein 10, breast cancer prognosis, tamoxifen therapy, response to treatment, survival

## Abstract

The human tissue kallikreins are secreted serine proteases, encoded by a group of homologous genes clustered in tandem on chromosome 19q13.3-4. Human kallikrein 6 and human kallikrein 10 are two new members of this family. Recently, we developed highly sensitive and specific immunofluorometric assays for human kallikrein 6 and human kallikrein 10, which allow for their quantification in tissue extracts and biological fluids. Both human kallikrein 6 and human kallikrein 10 are found to be down-regulated in breast cancer cell lines, suggesting that they may be involved in breast cancer pathogenesis and progression. In this study, we investigated the potential value of human kallikrein 6 and human kallikrein 10 as prognostic and predictive factors in breast cancer. We quantified human kallikrein 6 and human kallikrein 10 protein levels in 749 breast tumour cytosolic extracts and correlated this data with various clinicopathological variables and patient outcomes. Human kallikrein 6 and human kallikrein 10 are positively correlated with each other. Higher human kallikrein 6 and human kallikrein 10 protein levels are associated with younger age, pre-menopausal, status and tumours which are negative for oestrogen and progesterone receptors. No correlation was found between human kallikrein 6 and human kallikrein 10 levels and tumour size, grade, and nodal status. Survival analysis showed that neither human kallikrein 6 nor human kallikrein 10 are related to the rate of relapse-free and overall survival. In the analysis with respect to response to tamoxifen therapy, although human kallikrein 6 levels were not associated with tamoxifen responsiveness, higher levels of human kallikrein 10 were significantly associated with a poor response rate. This association remained significant in the multivariate analysis. Furthermore, higher human kallikrein 10 levels were significantly related with a short progression-free and post-relapse overall survival after start of tamoxifen treatment for advanced disease. Taken together, our results suggest that although human kallikrein 6 and human kallikrein 10 are not prognostic markers for breast cancer, human kallikrein 10 is an independent predictive marker for response of tamoxifen therapy.

*British Journal of Cancer* (2002) **86**, 1790–1796. doi:10.1038/sj.bjc.6600323 www.bjcancer.com

© 2002 Cancer Research UK

## 

The human tissue kallikreins are secreted serine proteases, encoded by a family of genes clustered in tandem on chromosome 19q13.3-4. All kallikreins share important similarities, including significant sequence homologies at both the DNA and protein level (Yousef and Diamandis, 2001). Initially, it was thought that in humans, this gene family consists of three members, including human kallikrein 1 (pancreatic/renal kallikrein), human kallikrein 2 (glandular kallikrein), and human kallikrein 3 (prostate specific antigen). Recently, 12 novel kallikrein-like genes were discovered in the same chromosomal region and their encoded proteins are considered as new members of the human kallikrein family. These new serine proteases were initially given different empirical names. A new nomenclature scheme has now been approved (Diamandis *et al*, 2000a) and the genes are known as KLK1… to KLK15 and the proteins as hK1… to hK15. This new nomenclature will be used in this study.

hK6 (also known as Zyme/Protease M/Neurosin) was independently cloned by three groups of investigators. Using polymerase chain reaction with degenerative primers for conserved regions in serine protease genes, hK6 was identified from Alzheimer's disease brain and the colon adenocarcinoma cell line colo 201, respectively (Little *et al*, 1997; Yamashiro *et al*, 1997). Meanwhile, by differential display, the same gene was cloned from a breast cancer cell line (Anisowicz *et al*, 1996). hK6 is a serine protease of 244 amino acids in length. Although it is highly expressed in the brain, it is also present in many other tissues and biological fluids, as shown by RT–PCR, immunoassay and immunohistochemistry (Yousef *et al*, 1999; Diamandis *et al*, 2000b; Petraki *et al*, 2001).

Human kallikrein 10 (hK10; also known as the normal epithelial cell-specific 1 gene) was discovered with subtractive hybridisation between normal and immortalised breast epithelial cell lines (Liu *et al*, 1996). This serine protease is composed of 276 amino acids. hK10 was found to be present in diverse tissues, such as breast, ovary, and prostate as well as in many biological fluids (Liu *et al*, 1996; Luo *et al*, 1998, 2001a).

The physiological functions of hK6 and hK10 are still not clear. Among all human tissue kallikreins, only hK1 has true kallikrein function, which, is defined as the ability to release kinins from kininogen (Clements, 1997; Yousef and Diamandis, 2001). All other kallikreins, presumably, act upon other substrates to mediate their physiological function. hK6 is predicted to have trypsin-like serine protease activity, whereas, hK10 is chymotrypsin-like. Several lines of evidence suggest that hK6 and hK10 actively participate in various pathological processes. Little *et al* (1997) showed that hK6 has amyloidogenic potential in the brain and may play a role in Alzheimer's disease. Others have demonstrated that hK6 is down regulated in aggressive forms of breast cancer, implicating that hK6 may be involved in breast cancer progression (Anisowicz *et al*, 1996). hK10 was also found to be down regulated in various cancer cell lines, including those from breast and prostate (Liu *et al*, 1996, Goyal *et al*, 1998). In testicular cancer, hK10 expression is significantly reduced or undetected in the tumour tissue compared to its adjacent normal tissue (Luo *et al*, 2001b). Furthermore, overexpression of hK10 can suppress tumour formation in nude mice, suggesting that it may function as a tumour suppressor (Goyal *et al*, 1998).

The best known marker for prostate cancer, PSA or hK3, is a member of the kallikrein family (Stamey *et al*, 1987; Oesterling, 1991). Since hK6 and hK10 seem to be involved in various diseases, and especially cancer, we speculated that their protein levels may change during disease initiation and progression and therefore, they may also be potential disease biomarkers. Recently, we developed highly sensitive and specific immunofluorometric assays for hK6 and hK10 (Diamandis *et al*, 2000b; Luo *et al*, 2001a) and provided evidence that these proteins are potential serum biomarkers for ovarian cancer (Diamandis *et al*, 2000c; Luo *et al*, 2001c) as well as prognostic markers for the disease (Luo *et al*, 2001d; Hoffman *et al*, 2002, submitted). We have also previously shown that both hK6 and hK10 are measurable in breast cancer cytosolic extracts (Diamandis *et al*, 2000b; Luo *et al*, 2001a). We have thus hypothesised that hK6 and hK10 may have a role as prognostic or predictive markers for breast cancer. In order to examine this hypothesis, we measured quantitatively levels of hK6 and hK10 in breast tumour cytosolic extracts and examined their relationship with clinicopathological variables including survival and response to tamoxifen treatment.

## MATERIALS AND METHODS

### Patients and tissue samples

Human kallikrein 6 and human kallikrein 10 levels were determined in cytosol preparations (as described below) from 749 primary invasive breast tumours collected between 1978 and 1990. Selection of samples was based on the availability of stored cytosol extracts (in liquid nitrogen), which remained after routine ER and PgR analyses and PSA as described earlier (Foekens *et al*, 1999). Of the 749 patients, 718 were eligible for analysis of relapse-free survival and overall survival according to the criteria described before (Foekens *et al*, 2000). Inoperable T_4_ tumours were not included. Patient tissues that were sampled after neoadjuvant treatment, or obtained from a biopsy specimen, were excluded. Patients who were referred to our institute more than 100 days after primary surgery and patients with distant metastasis at the time of primary surgery (M1 patients; staging according to the International Union Against Cancer TNM (tumour-node-metastasis) classification (Sherman and Hossfeld, 1990) were excluded from the analyses of relapse-free and overall survival. The latter patients were not necessarily excluded from the analysis of the response to first-line treatment with tamoxifen for advanced disease. In fact, these 31 patients were added to those who were eligible for our analysis of the response to tamoxifen therapy.

Analysis of relapse-free and overall survival was performed on 718 patients with primary operable breast cancer. Median age of these patients at the time of surgery was 55 years (range 24–89 years). Three hundred and six patients were pre-menopausal and 412 were post-menopausal at the time of primary surgery. Three hundred and ninety-five patients had undergone modified mastectomy and 323 patients, breast conserving treatment. Radiotherapy was given to 623 patients (87%): on the breast/thoracic wall in 503 patients and/or on the axilla in 272 patients, and/or parasternal and/or supraclavicular lymph nodes in 330 patients. T_1_ tumours (⩽2 cm) were present in 255 patients (36%), T_2_ tumours (>2–5 cm) in 360 patients (50%), T_3_ tumours (>5 cm) in 58 patients (8%), and operable T_4_ tumours in 45 patients (6%). Pathological examination was as described previously (Foekens *et al*, 1989) and the histological differentiation grade was coded as poor in 425 patients (59%), moderate in 118 patients (16%), well in 10 patients (1%), and unknown for 165 patients (23%). None of the 297 node-negative patients received systemic adjuvant therapy. Of the 421 node-positive patients, 196 had one to three nodes involved (27%), and 225 more than three nodes involved (31%). Of these patients, adjuvant chemotherapy (mainly cyclophosphamide/methotrexate/5-fluorouracil, CMF) was given to 138 patients (mainly pre-menopausal patients), whereas 62 patients received adjuvant hormonal therapy (mainly post-menopausal patients), either alone (51 patients) or in combination with chemotherapy (11 patients). All patients were examined routinely every 3–6 months during the first 5 years of follow-up and once a year thereafter. Of the 718 patients included, 420 (59%) showed evidence of disease during follow-up and count as failures in the analysis of relapse-free survival. Forty-eight patients (7%) died without evidence of disease and were censored at last follow-up in the analysis of relapse-free survival. Three hundred and eighteen patients (44%) died after a previous relapse. A total of 366 (48+318) patients (51%) were failures in the analysis of overall survival. The median follow-up period of patients alive (*n*=352) was 118 months (range, 16–211 months).

The following inclusion criteria were used for patients who received tamoxifen: patients with advanced disease who were treated with first-line tamoxifen therapy (40 mg day^−1^) and were not exposed to hormonal treatment at an earlier stage (hormono naive). Of the 420 patients of the 718 patients who relapsed, 211 subsequently received tamoxifen as first-line treatment. The remaining patients were treated by chemotherapy surgery, radiotherapy, other forms of systemic treatment, or died without further treatment. Following the same inclusion criteria, the subset of 211 patients was expanded with 31 patients who were previously excluded for analysis of relapse-free and overall survival. The median age of the total of 242 patients at start of treatment for advanced disease with tamoxifen was 61 years (range 33–87 years). Twenty-three per cent (*n*=56) of the patients were pre-menopausal, and 77% (*n*=186) post-menopausal. The first dominant site of disease was visceral in 89 patients, bone in 114 patients, and soft tissue in 39 patients. Seventeen patients (7%) had metastatic disease (M1 patients) at time of primary surgery. One-hundred and sixty-six patients (69%) had a disease-free interval (DFI) of >12 months between primary tumour removal and first recurrence. Only 51 patients had been treated with adjuvant polychemotherapy (CMF in 42 patients; FAC or FEC in nine patients). The median follow-up of patients still alive after start of tamoxifen treatment was 34 months (range 2–111 months). Thirty-one patients were still alive at the end of the present study, whereas 211 patients (87%) had died. On tamoxifen therapy, tumour progression occurred in 229 patients (95%) during follow-up. Of these patients, 151 were subsequently treated with one or more additional hormonal agents (mostly high-dose progestins), and to date, 126 patients have received systemic chemotherapy after the development of hormonal resistance (mainly CMF or FAC). The length of progression-free survival was defined as the time from the start of treatment for advanced disease until the start of next treatment because of progressive disease or until the time of intercurrent death. All patients were assessed by standard International Union Against Cancer criteria for objective response (complete remission, CR; partial remission, PR). Patients with no change for >6 months (stable disease, SDis) have a post-relapse overall survival similar to patients with partial remission (Ravdin *et al*, 1992; Foekens *et al*, 1994a). Therefore, for overall response, objective response and stable disease were combined (CR+PR+SDis).

### Assay of ER and PgR

Tumour tissues were stored in liquid nitrogen and pulverised in the frozen state with a microdismembrator as recommended by the European Organization for Research and Treatment of Cancer (EORTC) for processing of breast tumour tissue for cytosolic ER and PgR determinations (EORTC Breast Cancer Cooperative Group, 1980). The resulting tissue powder was suspended in EORTC receptor buffer (10 mM dipotassium chloride EDTA, 3 mM sodium azide, 10 mM monothioglycerol, and 10% v v^−1^ glycerol, pH 7.4). The suspension was centrifuged for 30 min at 100 000×**g** to obtain the supernatant fraction (cytosol). ER and PgR levels were determined by ligand binding assay or with enzyme immunoassay as described previously (Foekens *et al*, 1989). The cut-off point used to classify tumours as ER or PgR positive and negative was 10 fmol mg^−1^ protein.

### Quantification of hK6 and hK10 by immunofluorometric assays

The concentrations of hK6 and hK10 in the breast tumour cytosolic extracts were determined by immunoassays, as previously described (Diamandis *et al*, 2000b; Luo *et al*, 2001a). The hK6 assay is a sandwich-type immunoassay utilising mouse and rabbit anti-hK6 polyclonal sera. Briefly, mouse anti-hK6 polyclonal antiserum was first captured by sheep anti-mouse IgG-coated microtiter plates. Standards or samples were then added. Subsequently, rabbit anti-hK6 polyclonal antiserum and alkaline phosphatase conjugated-goat anti-rabbit IgG were sequentially applied to the plates. Finally, an alkaline phosphatase substrate was added and the signal was measured by time-resolved fluorescence. hK10 immunoassay is also a sandwich-type immunoassay, incorporating one monoclonal capture antibody and rabbit anti-hK10 polyclonal antiserum. In this assay, the hK10 monoclonal antibody was first directly coated on the polystyrene plates. Standards and samples were pipetted into each well, incubated and washed. Biotinylated rabbit anti-hK10 polyclonal antiserum was then added, incubated and washed. Finally, alkaline phosphatase conjugated-streptavidin was applied and the signal was detected as above, with time-resolved fluorescence. All tumour extracts were measured in duplicate. hK6 and hK10 concentrations in μg l^−1^ were converted to μg of hK6 or hK10 mg^−1^ of total protein to adjust for the amount of tumour tissue extracted.

### Statistics

The strength of the associations of hK6 and hK10 with continuous variables was tested with Spearman rank correlation (*r*_s_). The strength of the association of hK6 and hK10 (used as continuous variables) with other variables (used as grouping variable) was tested with the non-parametric Wilcoxon Rank-Sum test or Kruskal-Wallis test, followed by a Wilcoxon-type test for trend across ordered groups where appropriate. Survival probabilities were calculated by the actuarial method of Kaplan and Meier (1958). The logrank test was used to test for differences between survival curves. Both univariate and multivariate analyses were performed using the Cox proportional hazards model. The likelihood ratio test in the Cox regression models was used to test for differences and for interactions. The relationship of predictive factors with response to therapy was examined with logistic regression analysis. In our search for the best categorisation of hK6 and hK10, we have used isotonic regression analysis (Barlow *et al*, 1972; Foekens *et al*, 1994b) using the overall response rate as endpoint in those patients who received tamoxifen therapy for advanced disease. All computations were done with the STATA statistical package, release 6.0 (STATA Corp., College Station, TX, USA). All P values are two-sided.

## RESULTS

### hK6 and hK10 levels and patient and tumour characteristics

The levels of hK6 and hK10 in primary breast tumour cytosols ranged from 0–250 μg mg^−1^ of protein (median, 1.0 μg mg^−1^ of protein) and from 0–16.3 μg mg^−1^ protein (median, 39 ng mg^−1^ of protein), respectively. Table 1

**Table 1:**
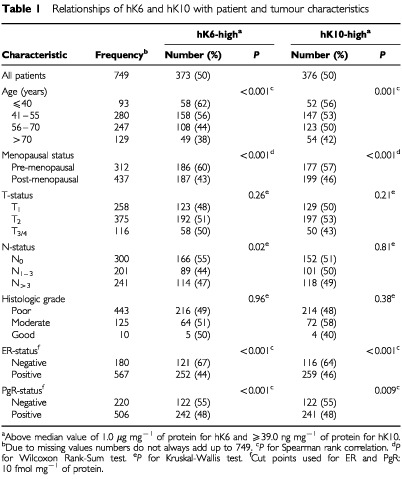
Relationships of hK6 and hK10 with patient and tumour characteristics

shows that younger and pre-menopausal patients more often had higher levels (above the median value) of hK6 and hK10, compared with older and post-menopausal patients. The Spearman rank correlations (*r*_s_) between the levels of hK6 and hK10 with age were −0.20 and −0.12, respectively. hK6 and hK10 levels were not significantly related with size or grade of the primary tumour. Tumours of node-negative patients more often had a higher level of hK6, while such an association was not present for hK10. The levels of hK6 and hK10 were negatively correlated with those of ER and PgR, with the steroid hormone-receptor negative tumours showing more often higher values (Table 1). The *r*_s_ was −0.35 for hK6 and ER, and −0.14 for hK6 and PgR. Those between hK10 and ER and PgR were −0.20 of −0.10, respectively. The levels of hK6 and hK10 were positively correlated with each other (*r*_s_=0.35, *P*<0.001).

### Relationship of hK6 and hK10 with relapse-free and overall survival

Of the 749 patients included in the study, 718 were eligible for analysis of relapse-free and overall survival. When studying hK6 or hK10 as log-transformed continuous variables, or as dichotomised variables at their median levels, in the analysis of relapse-free and overall survival, neither of them was significantly related with the rate of relapse in these 718 patients. In contrast, both in the analysis of relapse-free and overall survival, the traditional prognostic factors younger age (*P*=0.04 and *P*<0.001), larger tumour size (for both, *P*<0.001), the number of positive lymph nodes (for both, *P*<0.001), and poor tumour grade (for both, *P*=0.01), were all significantly associated with a worse prognosis. ER-positive or PgR-positive tumours were associated with a favourable prognosis, although the association of ER with relapse-free survival was not of statistical significance (*P*=0.56 and *P*=0.02 in the analysis of relapse-free survival, and *P*=0.05 and *P*<0.001 in the analysis of overall survival, respectively).

### Tamoxifen therapy: univariate analyses as a function of hK16 and hK10 status

Of the 242 patients who received tamoxifen as first-line treatment for advanced disease, 127 (52%) responded (eight CR, 27 PR, 92 SDis). The median duration of response in these responders was 14 months. Using logistic regression analysis, it was shown that older age and post-menopausal status were associated with a higher rate of response on tamoxifen treatment compared with younger age and pre-menopausal status (Table 2

**Table 2:**
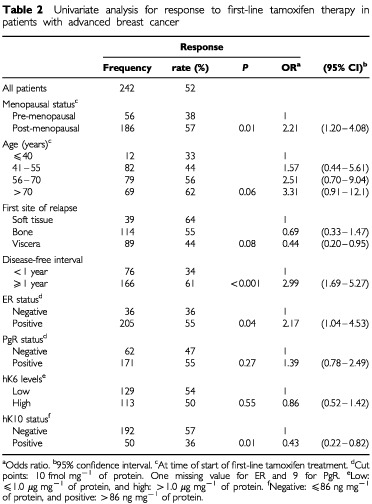
Univariate analysis for response to first-line tamoxifen therapy in patients with advanced breast cancer

). In patients with a DFI<12 months (34% response, OR set at 1) the fraction of responding patients was smaller than in patients with a DFI 1 year (61% response; OR, 2.99). Patients who relapsed to the viscera showed worse rate of response (OR, 0.44) compared with those who relapsed to the bone (OR, 0.69) or to soft tissues (OR, 1). Patients with ER-positive tumours had a more favourable response rate (55% response; OR, 2.17) than patients with ER-negative (36% response; OR, 1). When ER was analysed as a log-transformed continuous variable, higher levels were significantly associated with a higher response rate as well (*P*=0.003). In these patients, PgR and hK6 were not significantly associated with the rate of response when analysed as dichotomised variables (at 10 fmol mg^−1^ of protein and at the median level of 1.0 μg mg^−1^ of protein, respectively). When analysed as log-transformed continuous variable, higher levels of PgR were associated with a favourable response (*P*=0.008), but hK6 was not (*P*=0.24). hK6 was therefore not further considered in the present study. Adjuvant chemotherapy was not related to the rate of response to tamoxifen treatment. When analysing the relationship of continuous tumour hK10 levels with response, it appeared that increasing levels of hK10 were associated with a lower response rate (*P*=0.01). Using isotonic regression analysis, 86 ng hK10 mg^−1^ of protein was chosen as cut point to classify advanced breast cancer patients as hK10-positive and hK10-negative. Compared with the 192 hK10-negative patients (57% response (15% CR+PR, 42% SDis); OR, 1), the 50 hK10-positive patients showed a worse rate of response (36% response (12% CR+PR, 24% SDis), OR=0.43; *P*=0.01). Furthermore, the duration of response in the 192 hK10-negative patients was 9.0 months compared with 4.2 months for the 50 hK10-positive patients. Similarly the median overall survival time after start of tamoxifen treatment was longer for hK10-negative patients compared with hK10-positive patients (25 and 17 months, respectively). In Cox univariate regression analysis using continuous hK10 levels, higher levels significantly predicted a poor progression-free survival (*P*=0.04) and post-relapse overall survival (*P*=0.01). Similarly, when using hK10 as a dichotomised variable, hK10 positivity was associated with a rapid disease progression (Figure 1A

**Figure 1:**
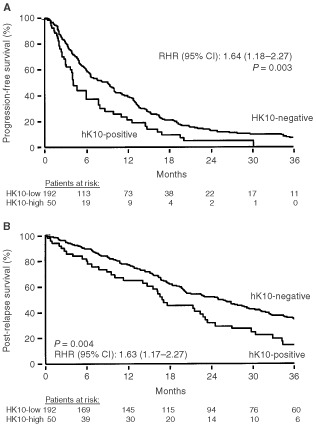
Progression-free survival (**A**) and post-relapse overall survival (**B**) as a function of hK10 status in advanced breast cancer patients treated with tamoxifen. Patients at risk are indicated. Cut point used for hK10, 86 ng mg^−1^ protein. RHR, relative hazards ratio. CI, confidence interval.

, *P*=0.003) and an early death (Figure 1B, *P*=0.004) after start of tamoxifen treatment.

### Tamoxifen therapy: multivariate analysis as a function of hK10 status

The independent relationship of hK10 status levels with the rate of response to tamoxifen treatment in advanced breast cancer was studied using multivariate logistic regression analysis. hK10 positivity was associated with a poor rate of response (OR, 0.47; 95% CI, 0.23-0.94; *P*=0.03). In addition to hK10, combined young age and post-menopausal status (*P*<0.01), a short DFI (*P*<0.001), and visceral metastasis (*P*=0.02) were independent factors which significantly predicted a poor response rate in the multivariate analysis. ER or PgR included either as a dichotomised variable or as log-transformed continuous variable, did not significantly contribute to the multivariate model. When hK10 was added as a log-transformed continuous variable instead of as a categorised variable to the multivariable model, the contribution of hK10 was of borderline statistical significance (*P*=0.06). There were no statistically significant interactions between hK10 status with any of the other variables included in the multivariate model for the rate of response to tamoxifen treatment.

## DISCUSSION

In this study, we measured hK6 and hK10 protein levels in breast tumour cytosols and found that they were positively correlated with each other, but negatively associated with ER and PgR. High hK10 is an independent predictor of response to tamoxifen therapy. hK6 and hK10, as well as other kallikreins, coexist in many tissues and biological fluids (Yousef and Diamandis, 2001; Diamandis *et al*, 2000b; Luo *et al*, 2001a). It has been reported that hK2 is able to activate hK3 by cleaving pro-hK3 (Kumar *et al*, 1997; Takayama *et al*, 1997). This observation suggests that kallikrein enzymes may participate in catalytic cascades and functionally interact with each other; for instance, one activates/inactivates another (Yousef and Diamandis, 2001). The positive correlation between hK6 and hK10 in breast tumour cytosols supports this hypothesis.

Due to the lack of knowledge on the physiological function of hK10 in breast tissue, the rationale underlying the reverse correlation between hK10 and ER and PgR is not clear. It is well documented that ER-negative tumours grow more aggressively and tend to metastasise more than ER-positive tumours (Andry *et al*, 1989; Winstanley *et al*, 1991). It is possible that hK10, a serine protease, may be associated with aggressiveness and metastatic potential of tumour cells. In another study, we showed that in ovarian cancer, high hK10 protein levels in tumour extracts correlate with late disease stage and poor survival (Luo *et al*, 2001d). These data support an association between hK10 overexpression and breast or ovarian cancer aggressiveness. The following two hypotheses may explain this association:

First, hK10 may participate in a pathway that involves promotion of cancer cell-growth. Previously, using a cell culture system, we found that hK10 mRNA level is up-regulated upon oestradiol and norgestrel stimulation in the breast cancer cell line BT-474 (Luo *et al*, 2000). Our further investigations demonstrated that hK10 was increased at both the mRNA and protein level not only by oestradiol and norgestrel, but also by other steroid hormones including dihydrotestosterone, dexamethasone and aldosterone in various breast cancer cell lines, such as MCF-7 and T-47D (unpublished data). Other investigators have reported steroid hormone receptor activation by non-steroidal ligands, including growth factors (Klocker *et al*, 1999). It is thus possible that hK10 may be up regulated by a variety of mechanisms in cancer. The enzymatic activity of hK10 may then be crucial for activation of downstream growth factors or receptors, as it has been shown for other serine proteases, including hK3, another member of the kallikrein family (Killian *et al*, 1993; Coughlin, 2000; Diamandis, 2000d). This activity may promote increased cell growth.

Second, hK10 may participate in a pathway that involves promotion of cancer cell metastasis. To metastasise, tumour cells turn on expression of factors that facilitate destruction of extracellular barriers (Liotta *et al*, 1991). Proteases are widely believed to be involved in these processes (Aznavoorian *et al*, 1993; Duffy, 1992). Therefore, the amount of proteases released by the primary tumour may reflect the ability of the tumour to spread. Overexpression of proteases has been reported in many cancers, such as of urokinase plasminogen activator (Look and Foekens, 1999), cathepsin D (Thorpe *et al*, 1989), and matrix metalloproteinase (Daidone *et al*, 1991). We speculate that hK10 may participate in a cascade reaction, which catalyses the breakdown of extracellular matrix, and thus, overexpression of hK10 may facilitate tumour migration.

It is well established that ER status correlates well with response to tamoxifen treatment. Still, a sizeable fraction of ER-negative patients do respond to tamoxifen, while not all of ER-positive patients respond. In this study, we found that hK10 levels can independently predict which patients will or will not respond to tamoxifen. These data are further reflected in both progression-free survival and post-relapse overall survival analysis (Figure 1). Interestingly, hK10 is the second member of this family whose increased expression correlates with poor response to tamoxifen therapy in breast cancer. We previously (Foekens *et al*, 1999) studied the other member, hK3 (PSA).

In summary, our study represents the first investigation showing that high hK10 protein levels are associated with low ER and PgR levels in breast tumours and that high hK10 levels in tumour tissue are independent predictors of poor response to tamoxifen therapy. The data presented herein should be considered as hypothesis generating and validation by an independent set of tumours is necessary. Our data may be useful for two purposes: (a) to shed more light on the biological mechanisms of tamoxifen resistance and the role of kallikreins in this process and (b) to identify additional biomarkers which have independent potential as predictors of therapeutic response in cancer.
